# Assumption of Constraining Force to Explain Distortion in Laser Additive Manufacturing

**DOI:** 10.3390/ma11112327

**Published:** 2018-11-19

**Authors:** Deqiao Xie, Jianfeng Zhao, Huixin Liang, Zongjun Tian, Lida Shen, Meng Xiao, Muhammad Naveed Ahsan, Changjiang Wang

**Affiliations:** 1 College of Mechanical and Electrical Engineering, Nanjing University of Aeronautics and Astronautics, Nanjing 210016, China; dqxie@nuaa.edu.cn (D.X.); hxliang@nuaa.edu.cn (H.L.); tianzj@nuaa.edu.cn (Z.T.); ldshen@nuaa.edu.cn (L.S.); 2 Nanjing Institution of Advanced Laser Technology, Nanjing 210038, China; nuaaxiaom@126.com; 3 Center of Excellence in Science and Applied Technologies, Sector H-11, Islamabad 44000, Pakistan; naveedahsan.ch@gmail.com; 4 Department of Engineering and Design, University of Sussex, Sussex House, Brighton BN1 9RH, UK; C.J.Wang@sussex.ac.uk

**Keywords:** distortion, stress, laser melting deposition, additive manufacturing

## Abstract

Distortion is a common but unrevealed problem in metal additive manufacturing, due to the rapid melting in metallurgy and the intricate thermal-mechanical processes involved. We explain the distortion mechanism and major influencing factors by assumption of constraining force, which is assumed between the added layer and substrate. The constraining force was set to act on the substrate in a static structural finite element analysis (FEA) model. The results were compared with those of a thermal-mechanical FEA model and experiments. The constraining force and the associated static structural FEA showed trends in distortion and stress distribution similar to those shown by thermal-mechanical FEA and experiments. It can be concluded that the constraining force acting on the substrate is a major contributory factor towards the distortion mechanism. The constraining force seems to be primarily related to the material properties, temperature, and cross-sectional area of the added layer.

## 1. Introduction

Additive manufacturing (AM) has become an established modern manufacturing technology, as it can quickly manufacture complex-shaped parts by using various materials including metal, resin, plastic, and ceramic [1,2,3,4,5]. Metal AM has brought new opportunities for designing novel structural materials, from designing complex geometries to controlling the microstructure (alloy composition and morphology) [6]. Most metal AM techniques [7], such as laser melting deposition (LMD), selective laser melting (SLM), electron beam melting (EBM), and wire arc additive manufacturing (WAAM), all undergo the “melt and solidification” process to achieve metal deposition. In other words, a metal is melted by a high-energy source (laser, electron beam, or arc) to form a molten pool and produce a large temperature gradient [8,9]. After the energy is removed, the molten pool is cooled. This is accompanied by another large temperature gradient and shrinkage of the molten pool. Undesired distortion is generated, reducing the precision of the additive manufactured part [10]. Additionally, cracks may also form in the manufactured metal parts during this period [11].

Literature surveys have shown various studies about distortion in metal additive manufacturing. J.-P. Kruth [12] performed post-process distortion measurements of parts manufactured using an SLM machine. He found that the distortion of a plate with an added layer is much larger than that of bare plates with the same laser scan pattern. Biegler [13] measured in-situ distortions in LMD walls using digital image correlation. The captured images revealed that the laser added wall showed a slight U-shape with the middle being bent downward in the Z-direction after cooling. Montevecchi [14] and Yu [15] also found symmetrical distortion of the substrate in WAAM and LMD processes, respectively. Gao’s investigation [16] showed that the distortion of the substrate along the Z-direction was much larger than that along the other two directions (X- and Y-directions) throughout the designed laser scanning patterns. Furthermore, the substrate distortion caused by laser fabrication was permanent and could not be recovered by heat treatment. Nickel [17] performed laser deposited metal experiments by using various deposition paths including a long raster, a short raster, and a spiral pattern. He found that the pattern has a significant effect on the distortion of the laser melted part. Yan found that island strategy can effectively decrease the maximum distortion in TC4 laser additive manufacturing [18]. Cao [19] has found that the preheating effectively mitigates the final distortion and the residual stress in EBM Ti-6Al-4V. Mukherjee found that the distortion could be enhanced by about 2.5 times when heat input doubled [20]. Denlinger [21,22,23] and Heigel [24,25] introduced a laser distance sensor into several metal AM techniques so as to measure the in-situ distortion of the substrate. They showed that the substrate experiences initial deflection in a downward direction, followed by an upward deflection. Additional dwell time allows additional cooling during the deposition process, which results in reduced distortion and residual stress in Inconel 625. However, for Ti-6Al-4V, decreasing dwell time results in significantly lower residual stress and distortion levels. Honnige successfully decreased the distortion and residual stress by post-deposition side-rolling [26]. In general, it can be concluded that Z-direction distortion is prominent and symmetrical. The distortion could be affected by various factors including deposition path, preheating, heat input, material properties, and post processing.

In order to investigate additional characteristics of distortion in metal AM, previous researchers established thermal-mechanical finite element analysis (FEA) models. The models revealed information about the distribution of temperature and residual stresses, as well as the overall distortion [6,27,28]. Zielinski [29] utilized a numerical model to investigate the distortion evolution of a bridge geometry part produced using an SLM process. He found that the newly deposited metal layers could dramatically transform the distortion distribution of previous deposited metal structures. Li [30] found that predicted residual stress in the length direction is a dominant factor for a part’s distortion. Afazov [31] proposed a novel simulation methodology to predict the distortion and then adjusted a CAD model for SLM. As a result, the experimental distortion was decreased from ±200 μm to about ±45 μm after compensation.

Researchers have focused on the regularity of distortion in metal AM and some influencing factors via experiments and thermal-mechanical FEA models. However, the mechanism of distortion in metal AM has not been fully revealed, nor have the critical factors. This study aims to explain the distortion in metal AM with a novel assumption of constraining force. Based on the assumption, the distortion is able to be controlled or adjusted with a quantitative guideline. Besides, the critical factors can be found out to be valued in studying and controlling the distortion. In this study, a static structural FEA model containing the constraining force will be used to show the distortion and stress distribution. These will be compared with the results of a thermal-mechanical FEA model and experiments, so as to validate the assumption of constraining force. In addition, the constraining force will be formulated. The relationships between the constraining force and the critical factors are able to be established and then validated. Finally, the exact values of constraining force will be calculated by comparing the static structural FEA and experimental results.

## 2. Materials and Methodology

### 2.1. LMD and AISI 316L

During this study, LMD was utilized because it generates obvious distortion, even when depositing just one track. It is vital to explore the intrinsic causes of distortion with little disturbance, such as the erratic heat accumulation in multi-track deposition. Another reason for using LMD is that it can be used to deposit repeatable tracks because laser is a kind of precise and stable energy.

In this experiment, austenitic stainless steel AISI 316L (EN 1.4404, Maite Powder, Beijing, China) was used, because it is a quotidian austenitic stainless steel that is commonly used in marine, energy, aerospace, and medical environments due to its excellent strength and corrosion resistance performances [32]. AISI 316L has been commonly researched in the context of metal AM [33,34,35].

### 2.2. Assumption of Constraining Force

**Figure 1:**
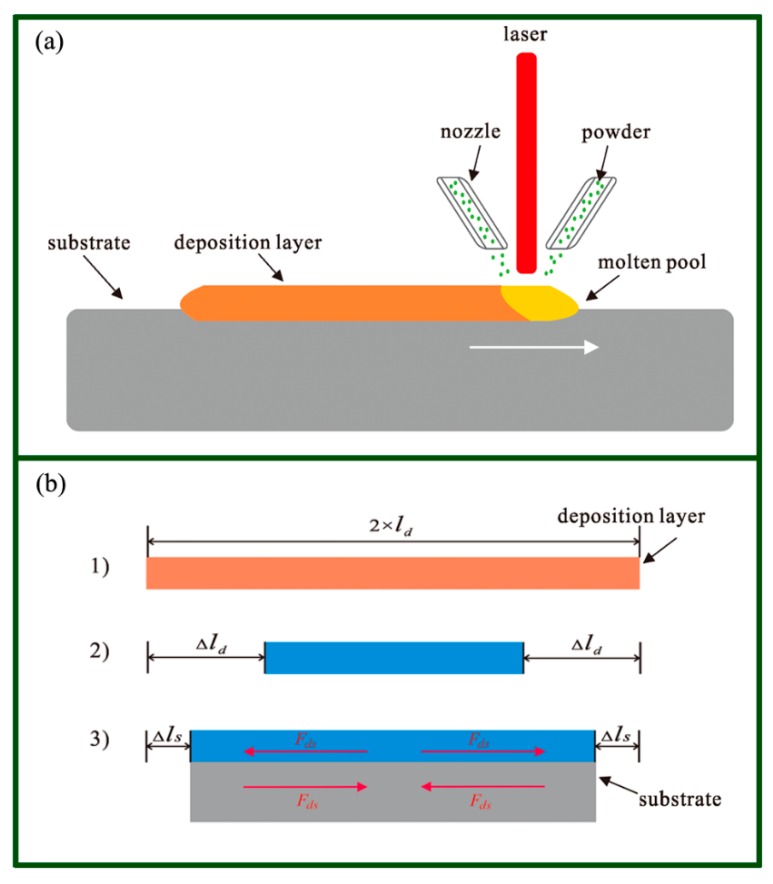
Assumption of the constraining force between a deposited layer and the substrate. (**a**) A schematic illustrating of laser melting deposition. (**b**) The assumption of constraining force *F_ds_*: (1) The length of deposited track at high temperature is 2 × *l_d_*. (2) The shrinkage of the deposited track at low temperature is 2 × Δ*l_d_* if there is no constraint. (3) The shrinkage of deposited track at low temperature was 2 × Δ*l_s_* if it is constrained by the substrate. From the view of elastic mechanics, the deposited track is stretched by forces from the substrate. The force caused by the constraint of the substrate was assumed to be *F_ds_*.

Figure 1a illustrates the laser converging on the substrate; this melted the substrate and the incoming powder, forming a molten pool. After the laser was removed, the molten pool cooled and solidified, forming a one-track deposition. The molten pool shrunk due to the temperature decline after the laser was removed. The deposited metal all underwent the same melting-cooling process in the single track LMD, so the deposited track was assumed to shrink uniformly. In Figure 1b, *l_d_* represents half the length of the deposited track at a high temperature. Δ*l_d_* is half the shrinkage of the deposited track at low temperature if there was no constraint. Nevertheless, the substrate at room temperature showed a little shrinkage. As a result, a balanced distribution of deformation was generated between the deposited track and the substrate. The deposited track was stretched by the substrate, while the substrate was compressed by the deposited track. Thus, the half shrinkage of the deposited track Δ*l_d_* could be restricted to Δ*l_s_* due to the substrate constraint. The interaction force along the longitudinal direction between the deposited track and the substrate was assumed to be *F_ds_*.

### 2.3. Static Structural FEA Model

**Figure 2:**
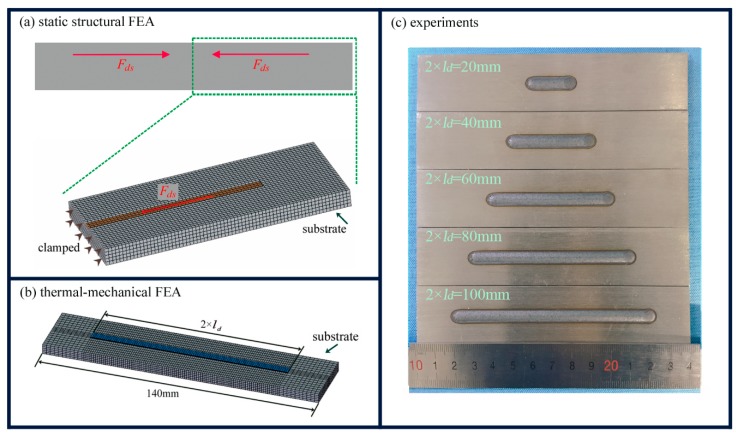
The three methods used in this study. (**a**) Static structural finite element analysis (FEA) model (half of the substrate). (**b**) Thermal-mechanical FEA model. (**c**) Single track samples with various deposition lengths.

Previous research found that the substrate and the deposited track have symmetrical distortion when the substrate is not clamped [13,14]. The distortion along the Z-direction is much larger than that along the other two directions [16]. As a result, it can be assumed that there is little Z-direction distortion on the middle plane along the scanning direction, while the maximum Z-direction distortion emerges at the free end of the substrate. This led us to establish a static structural FEA model in ANSYS (Figure 2a) which simulates half of the substrate and to set the middle plane as clamped. A 70 mm × 30 mm × 5 mm AISI 316L plate was assumed as the substrate, which experienced interaction force of *F_ds_* on an interaction area of *l_d_* (length) × 5 mm (width). The shrinkage of the deposited track Δ*l_s_* is negligible compared to the length *l_d_* at a higher temperature, so *l_d_* is also viewed as the numerical value of half the deposition length at room temperature. The assigned numerical values of *l_d_* were 10 mm, 20 mm, 30 mm, 40 mm and 50 mm. *F_ds_* was assumed to be 10 kN, as an attempt to estimate the value of the constraining force.

### 2.4. Thermal-Mechanical FEA Model

A thermal-mechanical FEA model was established to investigate difficult-to-measure information, e.g., temperature distribution and distortion. The thermal-mechanical FEA model was solved in two sequential steps. The first step applied a thermal analysis that determined the temperature gradient history during the laser deposition process. The second step was using a transient structural analysis that calculated the distortion from the temperature history [36].

The parametric design capabilities of the finite element code ANSYS enabled us to expediently change the sizes of the part geometry. In this paper, a 140 mm × 30 mm × 5 mm plate was assumed as the substrate of the LMD. A block of 2 × *l_d_* (length) × 5 mm (width) × 0.6 mm (height) was used as the deposited track, as seen in Figure 2b. The assigned numerical values of *l_d_* were 10 mm, 20 mm, 30 mm, 40 mm and 50 mm. Both the substrate and the deposited track shared the same thermal physical properties of the AISI 316L [37,38], as listed in Table 1. The model had a minimum size of 0.2 mm × 1 mm × 1 mm by using mapping mesh. The SOLID 70 element was used in the first step thermal analysis, while the SOLID 45 element was used in the second step stress analysis. The heat density distribution that the laser had on the contour plane was treated via the Gaussian model function [39]. The heat exchange took place between the build and substrate and the air [40].

The element “death and birth” technique was used to accomplish the simulation of laser powder melting and the deposition process [41]. In order to achieve more accurate results, the phase transformation was also taken into consideration in the model [42,43].

The main thermal-mechanical FEA parameters were listed as follows: Laser power 2000 W, absorption ratio 0.4, scanning speed 600 mm/min, laser spot diameter 5 mm, room temperature 20 °C, and cooling time 600 s.

### 2.5. LMD Experiments and Measurements

The experiments were performed on an LMD system consisting of a 6 kW TRUMPF disk laser with a working wavelength of 1064 nm, a KUKA robot, a GTI powder feeder, and a TRUMPF 3-nozzle deposition head. Spherical gas atomized AISI 316L powder, with diameters between 45 μm and 105 μm, was supplied to the deposition head by argon gas.

The 140 mm × 30 mm × 5 mm AISI 316L plates were used as the substrate for both single-track and single-track multi-layers of laser deposition. The main process parameters were follows: Laser power 2000 W, scanning velocity 600 mm/min, and powder feeding rate 6 g/min. The single-track samples with deposition lengths of 20 mm, 40 mm, 60 mm, 80 mm and 100 mm are shown in Figure 2c.

The distortion of the substrate was measured using a coordinate measuring machine from Leader Metrology Inc., Maryland, MD, USA. The displacement data of a 2 mm × 2 mm dot matrix on the back of the substrate were obtained. The measured data were used to create 3D models of the substrate in Origin 9.0 (OriginLab Corporation, Northampton, MA, USA), so as to show the distortion intuitively. The points at the centerline were extracted and plotted.

To measure the residual stress of the deposited section, an X-ray residual stress measurement [44] was taken using a PULSTEC-μX360 (PULSTEC Industrial, Shizuoka, Japan) with a Cr radiation source. The sin^2^ψ method was used for the stress measurement. The X-ray working voltage and working current were set to 30 kV and 1.5 mA respectively.

## 3. Results and Discussion

### 3.1. Distortion via Three Methods

**Figure 3:**
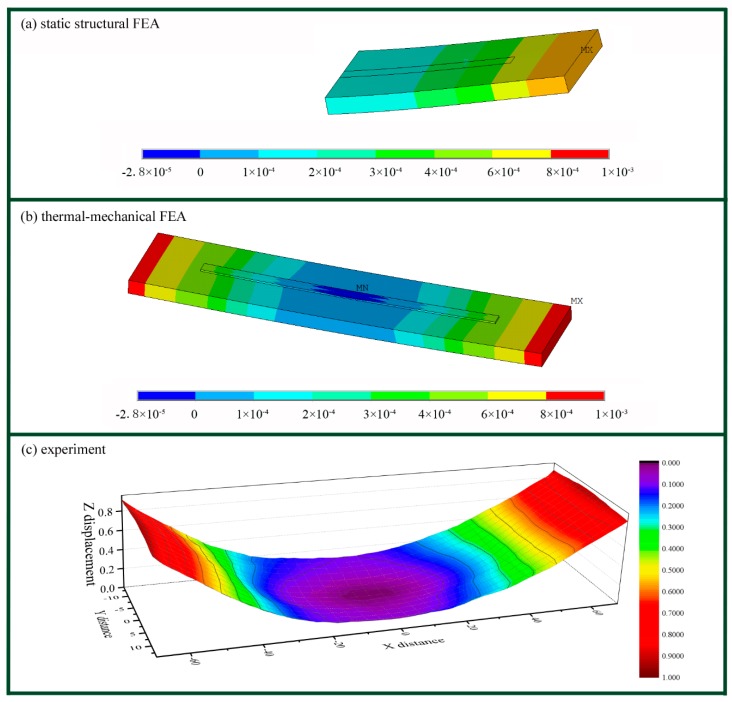
The distortion of the substrate as found by using the three methods. (**a**) The distortion of half of the substrate by as found by the static structural FEA when *l_d_* = 50 mm. (**b**) The distortion of the whole part as found by the thermal-mechanical FEA when *l_d_* = 50 mm. (**c**) The results of experimental distortion of the whole substrate when *l_d_* = 50 mm.

Figure 3 exhibits the distortion of the substrate as found using the above three methods, i.e., static structural FEA, thermal-mechanical FEA, and experiments. Both the thermal-mechanical FEA model (Figure 3b) and the experimental displacement measurement (Figure 3c) depicted symmetrical distortion of the substrate, together with peak Z directional displacement at the free ends of the substrate. Figure 3a shows that the static structural FEA model exhibited nearly the same distortion of the half substrate when compared to Figure 3b,c. This means that the static structural FEA model with the constraining force on the top surface partially simplified the analysis of the distortion caused by complex factors. The constraining force along the scanning direction, *F_ds_*, was likely the dominant factor that caused the distortion.

### 3.2. Distribution of Stresses

**Figure 4:**
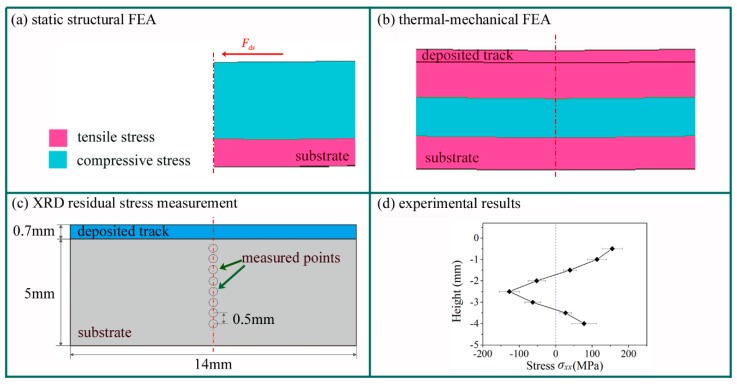
The distribution of stresses *σ_xx_* fouind via the three methods. (**a**) The stress distribution in the static structural FEA model (half of the substrate). (**b**) The distribution of residual stresses in the thermal-mechanical FEA model. (**c**) The measured points of XRD residual stresses tests. (**d**) The experimental residual stresses along the center line.

The consistency of the static structural FEA model and the other two methods was further verified by investigating the distribution of stress. The constraining force along the scanning direction primarily caused the distortion, so only the corresponding longitudinal stress *σ_xx_* was taken into consideration. In order to obtain a distinct comparison, the stress *σ_xx_* was divided into two types: Positive (tensile) and negative (compressive), regardless of its numerical value. Figure 4 demonstrates the distribution of the stress *σ_xx_* on the longitudinal section including the center-line of the width. As shown in Figure 4a, *σ_xx_* was negative at the upper section of the substrate and positive at the lower section, with a dividing line at a depth of about 3.4 mm from the top surface of the substrate. By balancing force and moment, the upper section of the substrate suffered compressive force, while the lower section experienced tensile force.

Figure 4b depicts a distribution of the stress *σ_xx_* similar to that in the thermal-mechanical FEA results, particularly for the depth of the dividing line at the lower section of the substrate. There was a zone of tensile stress in the upper section of substrate in the thermal-mechanical FEA model. The large temperature gradient of the laser heating affected the upper part of the substrate. When the laser was removed, shrinkage of deposited track was constrained by the surrounding cold zone of the substrate. As a result, both the upper substrate and the deposited track exhibited tensile residual stresses.

The residual stresses on the longitudinal section were measured via X-Ray DiffractionXRD. The positions of the measurement locations are shown in Figure 4c. Figure 4d shows that the longitudinal residual stress *σ_xx_* exhibits tensile-compressive-tensile change along the depth of substrate. Both Kruth [45] and Bendeich [46] found nearly the same change in residual stress with depth. The dividing line at the lower section of the substrate occurred between 3 mm and 4 mm. Therefore, the distribution of stresses given by static structural FEA was partly in accordance with the thermal-mechanical FEA and the experimental results, particularly at the depth of the dividing line.

### 3.3. Comparison of Displacement Curves

**Figure 5:**
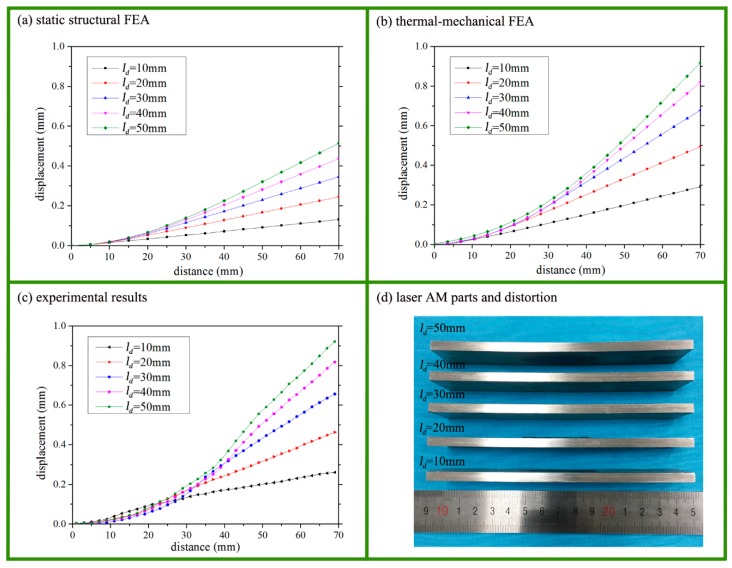
Displacement curves for the various *l_d_* values with the three methods. (**a**) The displacement curves for various *l_d_* values via the static structural FEA when the *F_ds_* is 10 kN. (**b**) The displacement curves for various *l_d_* values via the thermal-mechanical FEA. (**c**) Experimental displacement curves for the various *l_d_* values. (**d**) Experimental samples of the various *l_d_*.

As distortion was symmetrical, the Z-direction displacement of the half substrate along the length was extracted from the models and measured in experiments. Figure 5 depicts the displacement curves for various *l_d_* values using the above three methods. Figure 5a follows the same trends as those depicted in Figure 5b–d. We concluded that a longer deposition length *l_d_* leads to a more obvious distortion along with larger peak displacement. Examining the distribution of stresses allows a better understanding of this phenomenon. The tensile stress at the lower section of the substrate resulted in tensile strain while the compressive stress at the upper section of the substrate led to compressive strain. Thus, a bending angle occurred in the substrate. When the deposition length *l_d_* was increased, the bending angle accumulated and hence became larger. Therefore, the peak displacement rose, as depicted in Figure 5.

According to the above analysis, the results of the static structural FEA model were consistent with those of the thermal-mechanical FEA model and the experiments in terms of distortion, stress distribution, and displacement curves. Therefore, it may be deduced that the constraining force and its associated static structural FEA model could be applied to explain distortion in laser additive manufacturing.

## 4. Calculation of the Constraining Force

### 4.1. Expression of the Constraining Force

In order to investigate the key roles for determining distortion, a specific expression of the constraining force *F_ds_* was obtained mathematically. The deposited track was assumed to be an ideal elastic body, so as to obtain a quantifiable value of *F_ds_*. The deposited track was viewed as a rod with uniform tension along the longitudinal direction. Combined with Figure 1b, the constraining force *F_ds_* was expressed by Hooke’s law as follows:

(1) $$F_{ds}=\frac{\left(\Delta l_{d}-\Delta l_{d}\right)}{l_{d}}\cdot E_{d}\cdot A_{d}=\frac{\left(\Delta l_{d}-\Delta l_{d}\right)}{\Delta l_{d}}\cdot \frac{\Delta l_{d}}{l_{d}}\cdot E_{d}\cdot A_{d}=k_{ds}\cdot \frac{\Delta l_{d}}{l_{d}}\cdot E_{d}\cdot A_{d}=k_{ds}\cdot (\alpha \cdot \Delta T+\beta_{pt})\cdot E_{d}\cdot A_{d}$$

∆*l_d_* represents the shrinkage length of the deposited track at a low temperature if there was no constraint; ∆*l_s_* indicates the actual shrinkage length with constraint of the substrate; *E_d_* and *A_d_* are the Young’s modulus and the cross-sectional area of the deposited track at the room temperature *T_r_*, respectively. Each molten pool underwent the same heating-cooling process and was constrained by the same surrounding substrate, where the constraining coefficient could be defined as *k_ds_* = (∆*l_d_* − ∆*l_s_*)/∆*l_d_*. The coefficient is related to the material properties and the processing parameters. The ∆*l_d_* is composed of the thermal shrinkage *α_d_*∙∆*T* and the phase transformation shrinkage *β_pt_* [47]. The *α_d_* is the mean thermal expansion coefficient of the deposited track, while ∆*T* represents the temperature differences between the solidus temperature and the current temperature.

### 4.2. Calculation of the Constraining Force

In order to predict the distortion using the static structural FEA model, an accurate value of the constraining force *F_ds_* was calculated. For the AISI 316L, the mean thermal linear expansion coefficient *α_d_* is 20.02 × 10^−6^ with a solidus temperature of 1371 °C [48]. The room temperature was 20 °C.

Phase transformation during the solidification of austenitic steel depends deeply on the chemical composition of the alloy, especially Cr and Ni equivalences [49]. The chemical composition of AISI 316L (in wt.%) is 0.021% C, 1.79% Mn, 0.43% Si, 0.02% P, 0.001% S, 12.31% Ni, 17.43% Cr, 2.41% Mo, 0.16% Cu, 0.1% Co, 0.069% N, 0.00014% B and Fe balance. The Cr equivalence (*Cr_eq_*) and Ni equivalence (*Ni_eq_*) can be calculated using Equations (2) and (3).

(2) $$Cr_{eq}=[\%Cr]+[\%Mo]+1.5[\%Si]+0.5[\%Nb]=20.485\%$$

(3) $$Ni_{eq}=[\%Ni]+30[\%C]+0.5[\%Mn]=13.835\%.$$

According to Li [49], the solidification mechanism of AISI 316L should be FA mode as follows:

*L* → *L* + *δ* → *L* + *δ* + *γ* → *γ* + *δ* → *γ* (1.48 < *Cr_eq_*/*Ni_eq_* < 1.95).

*L*, *δ*, and *γ* represent liquid, delta-ferrite, and austenite, respectively. In other words, *δ*-ferrite may be primarily formed during solidification, and will transform into *γ*-austenite on successive cooling. Since the *δ* phase is body-centered cubic with a density of 68%, the volume will decrease when it is transformed to the face-centered cubic *γ* phase with a density of 74%. Thus, volume shrinkage was estimated to be 8.8%, while the homologous linear shrinkage *β_pt_* should be 2.78%. The shrinkage caused by *δ*-*γ* phase change was estimated in Kelly’s study on the initial solidification of 0.1 pct C steel [50].

**Figure 6:**
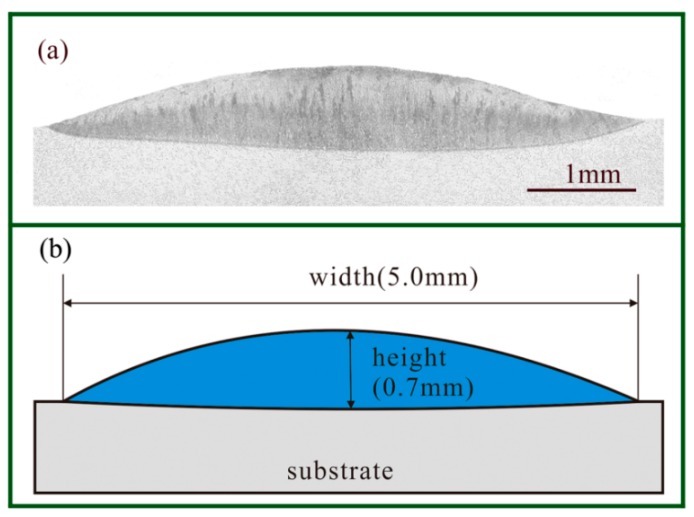
The cross-section of the deposited layer: (**a**) A metallographic microscope image and (**b**) a schematic diagram.

The Young’s modulus *E_d_* at room temperature was 1.96 × 10^11^ Pa which was obtained via the linear interpolation in Table 1. To investigate the cross-sectional area, the sample with a single track was cut via wire-EDM. The microstructure was observed on an OLYMPUSGX71 optical microscope after using the etchant of 5 mL HF (Hydrofluoric Acid). As shown in Figure 6a, the cross-sectional area had a width of 5.1 mm and a height of 0.7 mm. The cross-sectional area *A_d_* was about 2.38 mm^2^ obtained, via Photoshop software with statistics of pixels.

Based on Formula (1), the constraining force *F_ds_* can be calculated as follows:

(4) $$F_{ds}=k_{ds}\times [20.02\times 10^{-6}\times (1371-20)+2.78\times 10^{-2}]\times 1.96\times 10^{11}\times 2.38\times 10^{-6}=k_{ds}\times 2.54\times 10^{4}N$$

With the conjoint analysis of the static structural FEA, the constraining coefficient *k_ds_* could be calculated as follows:

(5) $$k_{ds}=d_{ex}/(d_{ss}\times 2.54)$$

Here, *d_ss_* denotes the peak displacement by the static structural FEA with a constraining force of 10 kN while *d_ex_* is the experimental mean peak displacement. Table 2 shows values of the constraining coefficient *k* related to the deposition length *l_d_*. The *k_ds_* decreases with the *l_d_*. A long deposition length may have a greater tendency of plastic deformation, which can release some regional constraint. The constraint could decrease to a certain extent, causing a decline of the constraining coefficient *k_ds_*.

## 5. Validation of Relationships between Distortion and Temperature and Cross-Sectional Area

### 5.1. Relationships between Distortion and Temperature

**Figure 7:**
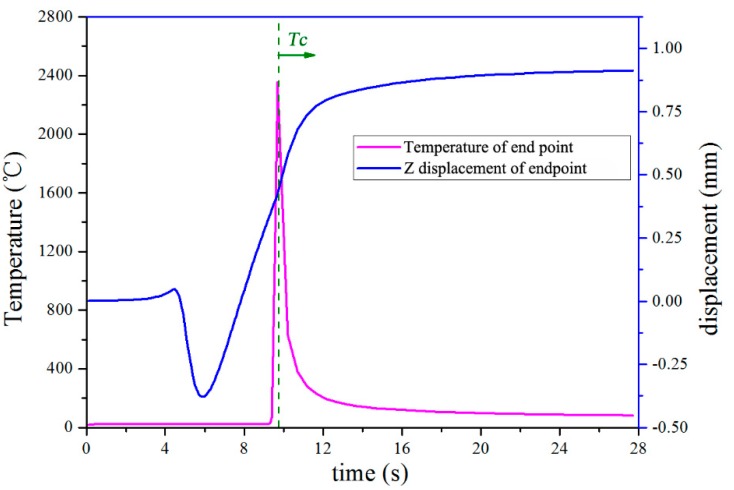
The changes in temperature and distortion over time.

The above studies showed that the distortion is likely to be caused by the constraining force *F_ds_*. Equation (1) shows that the constraining force *F_ds_* is related to material properties, temperature, and cross-sectional area. The experiment only used AISI 316L stainless steel, so changes in material properties are not discussed here. In order to investigate the relationship between distortion and temperature, the changes in temperature and distortion over time were extracted from one-track thermal-mechanical FEA model with a deposition length *l_d_* of 50 mm, as shown in Figure 7. The temperature of the end point on the top surface of the deposited track is adopted as a representation. The results demonstrate that the free end of the substrate was distorted downward, due to the thermal expansion of the laser heated zone. After the LMD was completed, the deposited track gradually contracted, causing the free end of the substrate to bend upward. It is worth noting that the positive displacement of the free end increased linearly with decreasing end point temperature from the time *T_c_* on, where *T_c_* means the beginning of cooling. The figure also shows that the temperature difference ∆*T* rarely changed after deposited track was cooled below about 200 °C. During the same period, the displacement displayed a little change. The above study revealed the relevance of distortion and temperature, especially in terms of the linear relationship between them during the cooling process. Denlinger [19] also concluded that changes in the temperature were correlated with observed distortion in experiments. Therefore, the relationship between distortion and temperature is partly verified.

### 5.2. Relationships between Distortion and Cross-Sectional Area

In terms of the cross-sectional area in Equation (1), Kruth’s study [12] elaborated that distortion of a plate with an added layer was larger than that of bare plates under the same laser melting conditions. Equation (1) was used to explain the phenomenon via the cross-sectional area as well. The cross-sectional area of the molten pool with added metal powder was larger than that of a molten pool with no added powder. The constraining force was also larger than that of a molten pool with no added powder, which made a significant difference in distortion. Haglund [51] found that the distortion is a function of melt volume and linear incremental displacement in half-overlapping pulsed laser melting, particularly for the first four pulses. R. J. Williams also found that various heights and widths of blocks in thermal-mechanical FEA made little difference on the prediction of distortion [52]. This allows the interpretation that the accumulated cross-sectional area increases the distortion, regardless of the partitions in the cross-section. As a result, the cross-sectional area plays an essential role in determining the distortion in metal AM.

**Figure 8:**
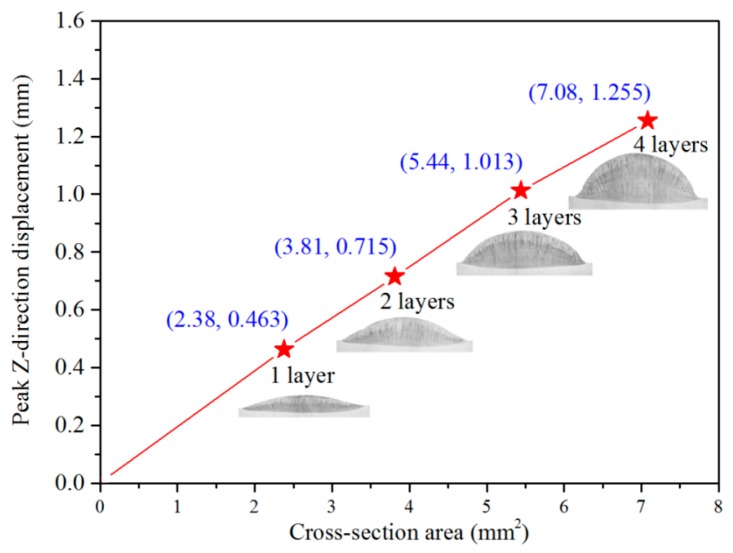
The evolution of distortion and its relationship with cross-section area.

To further validate the relationship between distortion and cross-sectional area, an experiment involving different layers of deposition was performed. Based on the same processing parameters as for the single-track deposition, the laser head was elevated by 0.7 mm layer by layer. A dwell time of 120 s between layers was applied to diminish the disturbance caused by heat accumulation. Figure 8 shows the peak Z-direction displacement and the cross-section area of various layers with *l_d_* of 20 mm. The distortion was enhanced as the metal was added layer by layer. The results also showed a distinct linear relationship between distortion and cross-sectional area, which also verified Equation (1).

## 6. Conclusions

The mechanism of distortion in metal additive manufacturing has been relatively unknown. In this study, a novel concept of constraining force was proposed to explain the phenomenon of distortion. The constraining force and a relevant static structural FEA model were validated by comparison with thermal-mechanical FEA and experiments. A mathematical expression was formulated to allow us to further understand the crucial factors in AM distortion. Our conclusions are as follows:

(i) The assumption of constraining force can be used to explain and better understand the distortion that occurs in metal AM.
(ii) Both the temperature and cross-sectional area play a critical role in determining the constraining force. In particular, the cross-sectional area accumulates during metal AM, causing almost linear increments in the constraining force and peak Z-directional displacement.

## Figures and Tables

**Table 1:** Thermal physical properties of AISI 316L stainless steel.

Temperature T/°C	Thermal Diffusivity λ/[W/(m·°C)]	Density ρ/(10^−3^ g/mm^3^)	Heat Capacity c/J/(kg·°C)	Linear Expansion Coefficient α/(10^−6^ °C^−1^)	Youngs Modulus E/(10^11^ Pa)
0	13.5	7.88	498.6	15.1	1.98
200	16.7	7.63	525.4	17.8	1.82
400	19.8	7.29	552.2	19.6	1.70
600	22.9	6.86	579.0	20.6	1.56
800	26.1	6.35	605.8	21.0	1.34
1200	32.4	5.04	659.4	21.4	0.58
1450	36.3	4.04	692.9	21.6	0.05

**Table 2:** Constraining coefficient values of the various deposition lengths.

Deposition Length *l_d_*/mm	Peak Displacement by Static Structural-FEA *d_ss_*/mm	Mean Peak Displacement of Experimental *d_ex_*/mm	Constraining Coefficient *k_ds_*	Constraining Force/kN
10	0.13	0.261	0.79	20.1
20	0.24	0.463	0.76	19.3
30	0.35	0.656	0.74	18.8
40	0.44	0.817	0.73	18.5
50	0.51	0.921	0.71	18.0
